# Exploring the Efficacy, Safety, and Clinical Implications of Deucravacitinib in East Asians with Psoriasis: A Narrative Review

**DOI:** 10.3390/jcm14051746

**Published:** 2025-03-05

**Authors:** Chul-Hwan Bang, Chul-Jong Park, Yoon-Seob Kim

**Affiliations:** 1 Department of Dermatology, Seoul St. Mary’s Hospital, College of Medicine, The Catholic University of Korea, Seoul 06591, Republic of Korea; 2 Department of Dermatology, Bucheon St. Mary’s Hospital, College of Medicine, The Catholic University of Korea, Seoul 14647, Republic of Korea

**Keywords:** deucravacitinib, East Asian, psoriasis, TYK2, dermatology

## Abstract

Deucravacitinib, a selective oral tyrosine kinase 2 (TYK2) inhibitor, has demonstrated strong efficacy in the treatment of moderate-to-severe psoriasis. It works through an allosteric mechanism to selectively inhibit TYK2, leading to the suppression of the IL-23/Th17/IL-17 axis and a reduction in key pro-inflammatory cytokines such as IL-17A, IL-17F, IL-22, and IL-23. This review focuses on the clinical implications of deucravacitinib in East Asian patients, highlighting its efficacy, safety, and differences in treatment outcomes compared to other populations. Data from pivotal trials such as POETYK PSO-3 and PSO-4, which included East Asian populations, demonstrated robust efficacy and safety profiles, often surpassing results observed in global trials like POETYK PSO-1 and PSO-2. Subgroup analyses and network meta-analyses further corroborate these findings, providing a comprehensive understanding of its therapeutic potential in this demographic. Factors such as lower body mass index, genetic predispositions, and environmental influences may contribute to these differences in response. The safety profile of deucravacitinib is favorable, with low rates of serious adverse events and stable laboratory parameters. This review underscores the need for further research to investigate the genetic, metabolic, and environmental factors that may influence treatment outcomes, aiming to optimize personalized treatment strategies for East Asian patients with psoriasis.

## 1. Introduction

Psoriasis, affecting approximately 2–3% of the global population, is a chronic inflammatory skin disease characterized by erythematous plaques and scaling [1]. Its pathogenesis involves a complex interplay of genetic, environmental, and immunological factors. Central to disease progression is the IL-23/Th17/IL-17 axis, where IL-23 supports Th17 cell differentiation and survival, leading to the production of pro-inflammatory cytokines such as IL-17A and IL-17F. These cytokines drive keratinocyte hyperproliferation and chronic inflammation, which are hallmark features of psoriasis [2].

Psoriasis in East Asian populations exhibits distinct clinical and genetic characteristics compared to other ethnic groups. Clinically, psoriasis in East Asians shows a tendency for lower prevalence and male predominance [3]. Additionally, certain subtypes, such as erythrodermic psoriasis, palmoplantar pustulosis, and generalized pustular psoriasis, may have a higher prevalence in East Asian countries [4,5,6]. Genetically, known risk allele frequencies are distinct between East Asians and other ethnicities. For example, the susceptibility gene HLA-Cw6 shows a lower prevalence in East Asians compared with other ethnicities [7]. Since the ethnic differences of psoriasis have not been fully elucidated, there may be additional clinical and genetic differences that are not yet documented. As psoriasis can be exacerbated by various environmental and internal exposome factors, these triggers may also differ in East Asian populations due to variations in environmental exposures and skin types [8,9,10]. Taken together, these clinical and genetic differences may influence the pathogenesis of psoriasis and treatment responses in East Asian patients.

Systemic treatments for moderate-to-severe psoriasis have evolved significantly over the past decades. These include conventional systemic disease-modifying antirheumatic drugs (csDMARDs) such as methotrexate and cyclosporine, biologics targeting specific immune pathways (e.g., TNF inhibitors, IL-17 inhibitors, and IL-23 inhibitors), and targeted synthetic DMARDs such as apremilast and deucravacitinib. Each of these therapies offers distinct mechanisms of action and therapeutic benefits [11]. Despite these advancements, the unmet need for safe, effective, and convenient oral treatment options persists, particularly for patients who are not candidates for biologics or prefer oral therapies.

Deucravacitinib, a novel oral treatment option for moderate-to-severe psoriasis, is classified as a selective allosteric inhibitor of tyrosine kinase 2 (TYK2). TYK2 is a key mediator in the signaling of cytokines central to psoriasis pathogenesis, including IL-23, IL-12, and type I interferons. By targeting TYK2, deucravacitinib offers a unique mechanism of action distinct from both biologics and traditional JAK inhibitors. Clinical trials have demonstrated its robust efficacy, with significantly higher Psoriasis Area and Severity Index (PASI) 75 and static Physician’s Global Assessment (sPGA) 0/1 response rates compared to placebo and apremilast at week 16 [12,13]. A network meta-analysis for long-term (44–60 weeks) efficacy of deucravacitinib and other biologic/nonbiologic treatments showed that the PASI 75 rate for deucravacitinib was 65.9%, which was higher than methotrexate (45.0%) and apremilast (48.4%), comparable to first-generation biologics such as adalimumab (62.8%) and ustekinumab (68.0%), and lower than second-generation biologics such as secukinumab (77.0%), ixekizumab (83.9%), bimekizumab (86.4%), guselkumab (87.8%), and risankizumab (91.6%) [14].

Historically, oral treatments for psoriasis included methotrexate and acitretin in the 1970s and 1980s, followed by cyclosporine in the 1990s. More recently, apremilast (2014) and dimethyl fumarate (2017) were introduced as targeted synthetic therapies [11]. Deucravacitinib, approved in 2022, represents a significant milestone in systemic therapy for moderate-to-severe psoriasis, with regulatory approval in multiple regions, including the United States, European Union, Japan, China, Taiwan, and Korea [15].

This review focuses on the efficacy and safety of deucravacitinib, particularly in East Asian populations. Both the characteristics of psoriasis and the effectiveness of treatments can differ across ethnic groups due to genetic, environmental, and cultural factors. Understanding these variations is essential for tailoring therapeutic approaches and achieving optimal clinical outcomes. Furthermore, this review explores potential factors contributing to differences in treatment responses among populations, offering insights to guide future research and clinical practice.

## 2. Mechanism of Action

Deucravacitinib is a first-in-class selective allosteric inhibitor of TYK2, a member of the Janus kinase (JAK) family. Unlike conventional JAK inhibitors that target the conserved ATP-binding catalytic domain (JH1), deucravacitinib binds to the structurally distinct regulatory pseudokinase domain (JH2) of TYK2 [16,17,18]. This selective binding mechanism distinguishes deucravacitinib from traditional JAK inhibitors, which often lack specificity and inhibit multiple JAK family members, leading to broader systemic effects.

Under basal conditions, the JH2 domain of TYK2 negatively regulates the adjacent catalytic (JH1) domain, preventing its activation. Deucravacitinib stabilizes the JH2 domain in an inactive conformation, thereby preventing JH1 activation and selectively inhibiting TYK2 signaling. This allosteric inhibition spares other JAK family members, such as JAK1, JAK2, and JAK3, minimizing off-target effects and reducing the risk of adverse events commonly associated with JAK inhibitors.

Preclinical studies have underscored the critical functions of TYK2 in immune and inflammatory processes. Tyk2-deficient mice exhibit impaired responses to interferon-alpha and interleukin-12 (IL-12), leading to diminished Th1 cell differentiation and function [19]. These findings highlight the importance of TYK2 in regulating the IFN/Th1- and IL-23/Th17/IL-17-signaling axes, which are central to psoriasis pathogenesis.

An in vitro study at clinically relevant doses demonstrated that deucravacitinib exhibits high specificity for TYK2, whereas traditional JAK inhibitors (e.g., tofacitinib, upadacitinib, and baricitinib) variably inhibit JAK1/2/3 but not TYK2 [20]. For example, deucravacitinib achieves 100-fold to 2000-fold in vitro selectivity for TYK2 over JAK1, JAK2, and JAK3 [21]. This high degree of selectivity enables targeted modulation of IL-23 and IL-12 cytokine activity, which are pivotal in the differentiation and proliferation of Th17 cells. In contrast, traditional JAK inhibitors inhibit multiple JAK family members, leading to broad immunosuppression. This lack of specificity can result in the unintended inhibition of vital immune functions and has been associated with increased risks of adverse events, such as infections and malignancies. The broad activity of traditional JAK inhibitors has been also linked to increased risks of cardiovascular events, thromboembolism, and malignancies, necessitating “black box warnings” from regulatory agencies [22,23,24]. In contrast, deucravacitinib’s selective inhibition of TYK2 is not associated with these risks, highlighting its potentially favorable safety profile.

TYK2 is integral to the IL-23/IL-17 axis, a key driver of psoriasis pathogenesis. IL-23 promotes the differentiation and survival of Th17 cells, which produce pro-inflammatory cytokines, including IL-17A, IL-17F, and IL-22. These cytokines perpetuate chronic inflammation and keratinocyte hyperproliferation, exacerbating psoriasis. By selectively inhibiting TYK2, deucravacitinib disrupts these signaling pathways, reducing cytokine production and alleviating psoriasis symptoms.

**Figure 1:**
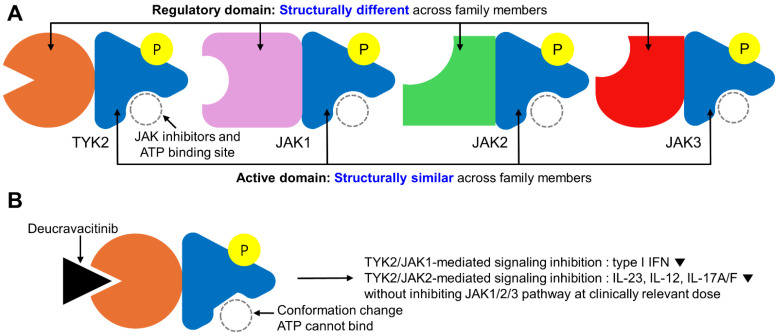
Mechanism of action of deucravacitinib and comparison with JAK inhibitors. (**A**) A schematic summarizing the differences between TYK2 and JAK1-3, as well as between deucravacitinib and traditional JAK inhibitors. (**B**) A schematic summarizing the mechanism of action of deucravacitinib.

The schematic figure illustrating the mechanism of action of deucravacitinib in the treatment of psoriasis is presented in Figure 1. This figure summarizes the selective targeting of TYK2 by deucravacitinib and its differentiation from traditional JAK inhibitors, providing a visual representation of its unique pharmacological profile.

## 3. Efficacy in East Asians

The pivotal POETYK PSO-1 (n = 666) and PSO-2 (n = 1020) trials established the significant efficacy of deucravacitinib in treating moderate-to-severe plaque psoriasis. These global, multicenter, double-blind, placebo- and active comparator-controled studies aimed to evaluate the efficacy and safety of deucravacitinib in diverse patient populations. Eligible participants were randomized to receive either a placebo, 6 mg once-daily deucravacitinib, or 30 mg twice-daily apremilast. Key inclusion criteria included a Psoriasis Area and Severity Index (PASI) score of ≥12, a static Physician’s Global Assessment (sPGA) score of ≥3, and a body surface area (BSA) involvement of ≥10%, ensuring a consistent baseline for evaluating moderate-to-severe disease severity [12,13].

Baseline demographics and disease characteristics were well-balanced across treatment groups in both trials. The mean age of participants was 46.6 years, and the average disease duration was 18.6 years. A subset of 18.4% had psoriatic arthritis (PsA), and 34.8% had prior exposure to biologic therapies, reflecting a population with significant disease burden and prior treatment experience [25].

At week 16, the PASI 75 response rates for deucravacitinib were significantly higher compared to both placebo and apremilast. Specifically, in PSO-1, 58.4% of patients achieved PASI 75 with deucravacitinib, compared to 35.1% with apremilast and 12.7% with placebo. In PSO-2, these rates were 53.0%, 39.8%, and 9.4%, respectively (*p* = 0.0004 for apremilast and *p* < 0.0001 for placebo) [4,5]. Similarly, response rates for sPGA 0/1 were significantly higher in the treatment group (53.6% for PSO-1 and 49.5% for PSO-2) compared to apremilast (32.1% for PSO-1 and 33.9% for PSO-2) and placebo (7.2% for PSO-1 and 8.6% for PSO-2) [4,5]. Improvements in patient-reported outcomes, such as the Psoriasis Symptoms and Signs Diary (PSSD) and Dermatology Life Quality Index (DLQI), further underscored the therapeutic benefits of deucravacitinib [26].

Long-term efficacy was demonstrated in the 3-year open-label extension of these trials. Continuous treatment with deucravacitinib maintained stable PASI 75 and PASI 90 response rates over time. By week 148, 58.4% of patients achieved PASI 75, and 35.6% reached PASI 90, highlighting the durability of treatment effects [27].

Despite the robust efficacy demonstrated in these global trials, the representation of East Asian populations was limited, with only 18.2% of participants in PSO-1 and 4.3% in PSO-2 identifying as East Asian [4,5]. To address this gap, subsequent studies focused on East Asian populations were conducted. These included the POETYK PSO-3 and PSO-4 trials, as well as a post-hoc subgroup analysis of Japanese participants from the PSO-1 trial [28,29,30].

In the phase 3 POETYK PSO-3 trial, conducted in East Asian patients from China, Taiwan, and South Korea, 220 participants with moderate-to-severe plaque psoriasis were randomized to receive either deucravacitinib 6 mg once daily or placebo for 16 weeks, followed by 52 weeks of open-label treatment. At week 16, significantly higher proportions of patients in the deucravacitinib group achieved PASI 75 (68.8% vs. 8.1%; *p* < 0.0001) and sPGA 0/1 (55.6% vs. 6.8%; *p* < 0.0001). These responses were sustained through week 52, underscoring the efficacy of deucravacitinib in East Asian populations [29].

The POETYK PSO-4 trial, an open-label study focusing on Japanese patients, further validated these findings. Among 63 participants with moderate-to-severe plaque psoriasis, 76.2% achieved PASI 75 by week 16, with sustained responses through week 52. Notably, the trial also included patients with generalized pustular psoriasis (n = 3) and erythrodermic psoriasis (n = 8), achieving PASI 75 response rates of 66.7% and 37.5%, respectively [30].

A subgroup analysis of Japanese participants in the PSO-1 trial revealed higher efficacy outcomes compared to the overall study population. PASI 75 and sPGA 0/1 response rates were 78.1% and 75.0%, respectively, compared to 58.4% and 53.6% in the global cohort. These findings suggest potentially greater efficacy in Japanese patients, although the reasons for these differences warrant further investigation [28].

A network meta-analysis of East Asian populations confirmed the robust efficacy of deucravacitinib for moderate-to-severe plaque psoriasis. PASI 75 and PASI 90 response rates at weeks 10–16 were 66% and 40%, respectively, significantly outperforming placebo and apremilast and showing comparable efficacy to biologic therapies such as adalimumab, certolizumab pegol, infliximab, ustekinumab, and tildrakizumab [15].

Real-world evidence further supports these findings. A 52-week study in Japan involving 104 patients with moderate-to-severe psoriasis demonstrated sustained clinical improvements, with 86.0%, 62.8%, and 25.6% of patients achieving PASI 75, 90, and 100, respectively. No significant safety concerns were reported, reinforcing the long-term utility of deucravacitinib [31]. Several smaller real-world studies involving fewer patients have also been published by the same author group [32,33,34].

Collectively, these data from clinical trials, subgroup analyses, and real-world studies highlight the robust efficacy of deucravacitinib in East Asian populations. Clinical outcomes in these populations often exceeded those observed in global trials, suggesting potential regional or genetic factors influencing treatment response. Key efficacy results are summarized in Table 1.

We further indirectly compared the statistical difference of merged PASI-75 and sPGA 0/1 responses at 16 weeks between the global pivotal clinical trials (POETYK PSO-1 and PSO-2, mainly Caucasians) and clinical trials in East Asians (POETYK PSO-3 and PSO-4). The PASI 75 response rates were statistically significantly higher in East Asian patients (70.3%; 147/209) compared to the global pivotal trials (55.2%; 465/843) (Chi-square test with Yates correction, *p* = 0.0001). Similarly, the sPGA 0/1 response rates were statistically significantly higher in East Asian patients (63.2%; 132/209) compared to the global pivotal trials (51.1%; 431/843) (Chi-square test with Yates correction, *p* = 0.0023). In spite of indirect comparisions, these findings demonstrate the significantly higher efficacy of deucravacitinib in East Asian patients compared to global pivotal trial participants.

## 4. Safety in East Asians

In the pooled analysis of the POETYK PSO-1 and PSO-2 trials, deucravacitinib demonstrated a favorable safety profile over 52 weeks. Exposure-adjusted incidence rates (EAIRs) for adverse events (AEs) were comparable across treatment groups, with low rates of serious AEs and discontinuations due to AEs. Nasopharyngitis and upper respiratory tract infection were the most common AEs associated with deucravacitinib and were numerically more frequent in deucravacitinib-treated patients than in those receiving placebo or apremilast. Serious infections occurred at an EAIR of 1.7 per 100 person-years, while herpes zoster was reported at 0.8 per 100 person-years. The EAIRs of AEs of interest, such as serious infections, major adverse cardiovascular events, and malignancies, were not significantly higher compared to placebo and apremilast. However, AEs of interest such as acne and folliculitis were significantly more frequent with deucravacitinib. Laboratory parameters remained low and stable throughout the treatment period [35].

In the POETYK PSO-LTE trial, a 3-year open-label extension of PSO-1 and PSO-2 trials, the safety profile remained consistent, with a decrease in EAIRs for AEs from the 1st to the 3rd year. Specifically, EAIRs for AEs decreased from 229.2 per 100 person-years in the 1st year to 144.8 per 100 person-years in the 3rd year, demonstrating an improved safety profile over time. The most common AEs were nasopharyngitis and upper respiratory tract infections, with serious AEs and discontinuations remaining rare and stable over time [27]. Although detail data were not yet published, recent 5-year data from the POETYK PSO-LTE trial indicate that deucravacitinib continues to demonstrate consistent safety and sustained response rates in moderate-to-severe plaque psoriasis, with 46.3% of patients achieving PASI 90 at year 5 and no new safety concerns identified [36].

The POETYK PSO-3 trial, including East Asian patients from China, Taiwan, and South Korea, confirmed the safety profile in this population. Common AEs were nasopharyngitis and upper respiratory tract infections, with serious AEs and discontinuations being rare [29]. The POETYK PSO-4 trial, focused on Japanese patients, showed similar findings, with nasopharyngitis, headache, and acne being common AEs and no severe infections or malignancies reported [30]. A subgroup analysis of Japanese patients from the PSO-1 trial also showed safety outcomes consistent with the overall study population [28].

Real-world studies further support the safety of deucravacitinib in East Asian populations. A 52-week real-world study conducted in Japan on 104 patients with moderate-to-severe psoriasis reported no significant changes in laboratory inflammatory indices, and no severe or life-threatening adverse events were observed [31]. Additionally, several smaller real-world studies involving fewer patients have been published by the same author [32,33,34]. These studies consistently reported low rates of AEs, with no significant safety concerns identified during the observation periods.

Further analysis highlights that the safety profile of deucravacitinib remains stable across various East Asian subgroups, regardless of demographic or clinical variations. The absence of significant increases in serious infections, malignancies, or cardiovascular events across clinical trials and real-world studies underscores the favorable risk–benefit ratio of deucravacitinib in this population.

Overall, the data from the POETYK PSO-3 and PSO-4 trials, along with the subgroup analysis from the PSO-1 trial and supporting real-world evidence, demonstrate that deucravacitinib is safe and well-tolerated in East Asian populations. The safety results from the key clinical trials are summarized in Table 2, providing a comprehensive overview of adverse event rates and their implications for clinical practice.

## 5. Potential Underlying Factors for Improved Outcomes in East Asians

Several factors may contribute to the observed differences in treatment outcomes of deucravacitinib for East Asian populations with moderate-to-severe plaque psoriasis. These include low body mass index (BMI), genetic predispositions, and environmental influences, all of which can collectively affect disease progression, therapeutic response, and drug metabolism.

Low BMI is a distinctive characteristic often observed in East Asian populations and may significantly influence the pharmacokinetics of psoriasis treatments. Variations in drug absorption, distribution, metabolism, and excretion associated with lower BMI could potentially lead to improved efficacy and altered safety profiles of medications like deucravacitinib. Obesity, on the other hand, is well-documented to be associated with a pro-inflammatory state, contributing to heightened disease severity and poorer therapeutic outcomes [37,38,39,40]. Studies in animal models, such as C57BL/6 mice, have shown that obesity exacerbates psoriasis through mechanisms involving increased secretion of pro-inflammatory cytokines, such as IL-6 and TNF-α, as well as an expansion of the Th17 cell population [41]. These findings underscore the interplay between metabolic factors and immune dysregulation in psoriasis pathogenesis.

Moreover, clinical evidence suggests that patients with lower BMI tend to exhibit superior treatment responses to biologics for psoriasis when compared to those with higher BMI [42,43,44,45]. Notably, the mean body weight of participants in East Asian trials of deucravacitinib was significantly lower (e.g., PSO-3: 26.3 kg/m^2^; PSO-4: 27.7 kg/m^2^) compared to participants in global trials (e.g., PSO-1: 29.8 kg/m^2^; PSO-2: 31.0 kg/m^2^), potentially accounting for the enhanced efficacy observed in East Asian cohorts.

Interestingly, other biologics exhibit varying efficacy patterns across ethnic groups, irrespective of BMI differences. For instance, guselkumab demonstrated comparable efficacy between Asian and non-Asian populations with moderate-to-severe psoriasis, despite the lower BMI of the Asian cohort (26.6 kg/m^2^) compared to the non-Asian cohort (30.1 kg/m^2^) [46]. In contrast, risankizumab displayed the lowest PASI reduction at week 100 in East Asian populations relative to other ethnicities despite the relatively lower body weight in East Asians [47]. These disparities highlight the complexity of ethnic-specific responses and suggest the involvement of additional factors beyond BMI in influencing therapeutic outcomes.

Genetic factors are pivotal in determining both the pathogenesis of psoriasis and the variability in treatment responses. Previous studies, particularly those focusing on anti-TNF-α therapies, have demonstrated the significant role of genetic predispositions in modulating drug sensitivity and efficacy. Genome-wide association studies (GWASs) in East Asian populations have identified both shared and unique genetic associations with psoriasis compared to European populations. While loci such as *IL12B* are commonly implicated across ethnicities, East Asian-specific variants, including *ERAP1* and *ZNF816A*, have been identified. These variants, absent in European cohorts, may influence immune response pathways, potentially enhancing the efficacy of IL-23 inhibitors like deucravacitinib by modulating antigen processing or inflammatory cascades [48,49,50,51,52].

Furthermore, genetic polymorphisms affecting drug-metabolizing enzymes and transporters may contribute to inter-individual and inter-ethnic variability in drug response. Although pharmacokinetic studies specific to deucravacitinib in various ethnicities are not yet available, understanding these genetic differences is crucial. Future pharmacogenomic and pharmacokinetic studies are needed to elucidate how these variations may influence drug absorption, distribution, metabolism, and excretion, ultimately affecting clinical outcomes.

Environmental factors also play a crucial role in shaping disease outcomes and therapeutic responses. Differences in diet, air quality, ultraviolet exposure, and traditional medicine practices are particularly relevant in East Asia. For instance, East Asian diets, characterized by a high intake of antioxidants and anti-inflammatory compounds, may synergize with pharmacological treatments to improve therapeutic outcomes. Additionally, reduced ultraviolet exposure due to lifestyle or geographic factors may influence disease severity and response to phototherapy or systemic treatment [53,54,55].

Collectively, these factors underscore the importance of considering ethnic-specific characteristics when developing and optimizing treatment strategies for East Asian patients with psoriasis. A comprehensive understanding of the interplay between BMI, genetics, and environmental influences will enable clinicians to tailor treatment approaches, achieve better disease control, and minimize adverse events in this population.

## 6. Limitations and Future Directions

The most significant limitation of our review is the lack of direct head-to-head clinical trials comparing deucravacitinib in psoriasis patients of different ethnicities. Such trials are crucial for drawing definitive conclusions regarding the higher efficacy of deucravacitinib in East Asians. Furthermore, the major ethnicities in pivotal clinical studies of deucravacitinib are Caucasians and Asians; for example, ethnicities for the pivotal trials were as follows: POETYK PSO-1 (White 80.2%, Asian 18.2%, and others 1.8%) and PSO-2 (White 91.7%, Asian 4.3%, and others 3.0%). There is limited data on the impact of deucravacitinib in African and Hispanic populations, warranting further investigation.

In recent years, with the advent of biologics, treatment has demanded a significant economic burden on psoriasis patients [56,57,58]. Cost-effectiveness data for deucravacitinib are currently limited due to its recent approval. However, due to its oral administration and the potential reduction in healthcare costs compared to biologics, deucravacitinib could offer a more cost-effective treatment option. Economic evaluations comparing the cost-effectiveness of deucravacitinib with existing conventional immunosuppressants (e.g., methotrexate, cyclosporin), biologics, and JAK inhibitors are needed to determine its value proposition in different healthcare settings. Furthermore, factors such as drug accessibility and insurance coverage vary across countries and will influence the affordability and overall cost-effectiveness of deucravacitinib. Understanding these factors is essential for optimizing the use of deucravacitinib and ensuring that patients have access to effective and affordable treatment options.

Treating psoriasis in geriatric patients can be challenging due to comorbidities, polypharmacy, and physical impairments [59]. Furthermore, psoriasis is well-known to be associated with an increased risk of various cardiovascular and endocrine comorbidities [60]. These factors can impact the safety outcomes of deucravacitinib in geriatric patients or those with multiple comorbidities. For example, age-related changes in drug metabolism, the presence of multiple health conditions, and potential interactions with other medications can affect the drug’s safety profile. Preliminary findings suggest that deucravacitinib’s selective inhibition of TYK2, with minimal off-target effects on other JAK family members, may offer a favorable safety profile compared to traditional JAK inhibitors. However, current data on deucravacitinib’s safety in these populations are limited, and further research is needed to provide comprehensive guidelines. Clinicians should consider individual patient characteristics when prescribing deucravacitinib to vulnerable populations, ensuring close monitoring for potential adverse events and making necessary dosing adjustments.

## 7. Conclusions

In this review, we examined the efficacy and safety of deucravacitinib in treating moderate-to-severe psoriasis, with a particular focus on its favorable outcomes in East Asian populations. Early findings suggest that deucravacitinib holds promise as a valuable therapeutic option for psoriasis management in this subgroup. The unique combination of genetic, environmental, and metabolic factors likely contributes to the enhanced response observed in East Asians.

The high efficacy of deucravacitinib observed in clinical trials for East Asian patients underscores its significant promise as a pivotal therapeutic option in this population. However, to fully understand the real-world impact of deucravacitinib, further real-world studies are needed. Additionally, real-world studies should assess treatment adherence, patient-reported outcomes, and clinicians’ experiences in East Asian healthcare settings. By capturing these aspects, real-world studies will provide valuable insights into the long-term efficacy and safety of deucravacitinib, helping to optimize its use in clinical practice and improve patient outcomes.

While the current evidence highlights the potential benefits of deucravacitinib, further research is essential to elucidate the long-term outcomes and the underlying mechanisms driving these ethnic-specific differences. Investigating the role of pharmacogenomic and pharmacokinetic variations, as well as lifestyle and environmental influences, will be critical for a deeper understanding of the underlying causes of these ethnic differences.

Clinicians should remain vigilant in reviewing emerging data to optimize the use of deucravacitinib and other therapies for East Asian patients. Future studies should prioritize pharmacogenomic profiling to identify predictive genetic markers for treatment response, enabling a more tailored and effective approach to managing psoriasis. By integrating these insights, we can move closer to achieving precision medicine in dermatology, ultimately improving patient outcomes and quality of life.

## Figures and Tables

**Table 1:** Summary of efficacy results from the key clinical trials of deucravacitinib.

Ref. (Year)	Study Name	Study Methods (Patients (n))	16 wk PASI 75 (Versus Placebo)	52 wk PASI 75	16 wk sPGA 0/1 (Versus Placebo)	52 wk sPGA 0/1
[12] (2023)	POETYK PSO-1	52-week double-blinded, phase 3 study (n = 666)	58.4% (12.7%)	65.1%	53.6% (7.2%)	52.7%
[13] (2023)	POETYK PSO-2	52-week double-blinded, phase 3 study (n = 1020)	53.0% (9.4%)	N/A	49.5% (8.6%)	N/A
[29] (2025)	POETYK PSO-3 (East Asian)	52-week, double-blinded, phase 3 study (n = 220)	68.8% (8.1%)	71.0%	55.6% (6.8%)	51.0%
[30] (2024)	POETYK PSO-4 (Japanese)	52-week, open-label, phase 3 study (n = 63)	76.2% (N/A)	82.5%	82.5% (N/A)	79.4%
[28] (2023)	POETYK PSO-1 Subgroup (Japanese)	Japanese subgroup from PSO-1 study (n = 66)	78.1% (11.8%)	75.0%	75.0% (11.8%)	62.5%
[15] (2024)	NMA (East Asian)	Systematic review and NMA of electronic databases	66% (vs 6%)	N/A	N/A	N/A
[33] (2025)	RWE from Japan	Single-center, retrospective study (n = 70)	69% (N/A)	N/A	N/A	N/A

N/A (not available); NMA (network meta-analysis); PASI (psoriasis area and severity index); PGA (physician global assessment); RWE (real-world evidence).

**Table 2:** Summary of safety results from the key clinical trials of deucravacitinib.

Ref. (Year)	Study Name	Study Methods (Patients (n))	Any AE (EAIR/100PY) (Versus Placebo)	Serious AE (EAIR/100PY) (Versus Placebo)	Tx-Related AE (EAIR/100PY) (Versus Placebo)	AEs Leading to Discontinuation (EAIR/100PY) (Versus Placebo)
[12] (2023)	POETYK PSO-1	52-week double-blinded, phase 3 study (n = 666)	211.8 (202.5)	7.5 (19.2)	33.1 (45.2)	3.3 (14.7)
[13] (2023)	POETYK PSO-2	52-week double-blinded, phase 3 study (n = 1020)	242.4 (221.5)	4.3 (2.5)	40.2 (42.4)	5.2 (8.0)
[25] (2024)	POETYK PSO-1/ PSO-2 pooling	52-week analysis of PSO-1 and PSO-2 trials (n = 1683)	2292 (217.4)	5.7 (5.7)	N/A	4.4 (9.3)
[27] (2025)	POETYK PSO-1/ PSO-2 pooling	3-year analysis of PSO-1 and PSO-2 trials (n = 1519)	144.8 (N/A)	5.5 (N/A)	N/A	2.4 (N/A)
[29] (2025)	POETYK PSO-3 (East Asian)	52-week, double-blinded, phase 3 study (n = 220)	319.9 (364.7)	6.5 (4.5)	100.3 (74.1)	2.7 (0)
[30] (2024)	POETYK PSO-4 (Japanese)	52-week, open-label, phase 3 study (n = 123)	172.6 (N/A)	6.7 (N/A)	N/A	3.3 (N/A)
[28] (2023)	POETYK PSO-1 Subgroup (Japanese)	Japanese subgroup from PSO-1 study at 52-week (n = 66)	336.8 (321.0)	11.7 (0)	34.1 (64.0)	4.5 (19.7)
[33] (2025)	RWE from Japan *	52-week, single-center, retrospective study (n = 70)	N/A	0% (0/104)	23.1% (24/104)	1.9% (2/104)

AE (adverse event); EAIR (exposure-adjusted incidence rates); N/A (not available); PY (person-year); RWE (real-world evidence); Tx (treatment). * Because EAIR/100PY value is not presented for the study, proportion of patients (%) was shown.
